# The Use of Fluoroscan in Hand Clinic During the Covid Pandemic to Optimise Conservative Treatment

**DOI:** 10.7759/cureus.29494

**Published:** 2022-09-23

**Authors:** Mohamed Nagy, Neil Ashwood, Amr Abouelela, Suraj Suryawanshi, Gur Aziz Singh Sidhu, Christos Kitsis

**Affiliations:** 1 Trauma and Orthopaedics, Cairo University Hospitals - Kasr Alainy, Cairo, EGY; 2 Trauma and Orthopaedics, University Hospitals of Derby and Burton, Derby, GBR; 3 Trauma and Orthopaedics, University of Wolverhampton, Wolverhampton, GBR; 4 Trauma and Orthopaedics, Royal Berkshire Hospital, Reading, GBR; 5 Orthopaedics, Max Saket Hospital, New Delhi, IND; 6 Trauma and Orthopaedics, University Hospital Lewisham, Lewisham and Greenwich (LG) NHS Trust, London, GBR

**Keywords:** clinic, hand, pandemic, covid 19, fluoroscan

## Abstract

Introduction

The study assessed the use of Fluoroscan (Hologic, Inc., Marlborough, MA) in hand clinic as advised by the British Orthopaedic Association (BOA) during the COVID-19 pandemic to facilitate treatment of fractures requiring manipulation and reduce admissions to evaluate if this should be embedded in practice permanently.

Method

Eighty-three wrist and hand fractures requiring manipulation were identified between April 2020 and March 2021. Demographics, mechanism of injury, timing of intervention, radiological outcome, further intervention and functional assessment by QuickDASH scoring were recorded.

Results

Sixty-eight cases were manipulated within the first week of fracture, simple pain control measures were used, and dose area product (DAP) averaged 1.3 Gy cm^2^ well below the dose limit set by the trust. Satisfactory fracture reduction was achieved in 59 cases avoiding admission. Further surgical intervention was offered to 24 patients: five re-manipulated while 19 had operation, all with a good functional outcome.

Conclusion

Fluoroscan use in fracture clinics achieved effective fracture control in 77% of cases. The use of Fluoroscan avoided admissions for surgery during the pandemic and lengthy clinic visits, four out of five did not need admission.

## Introduction

Mobile fluoroscopy was first introduced in trauma surgery in the early 1950s, facilitating fracture fixation in real-time without delay and improving outcomes, although this involved radiation exposure for the patient, surgeon, and allied health professionals assisting in the operative room [1].

The utilization of mini-C-arm fluoroscopic devices to treat and evaluate pathologies in the hand or wrist has increased throughout the years due to improvements in the image quality of these devices [2]. The mini-C-arm brings several advantages, including usability, often being operated by a surgeon with appropriate training rather than a radiographer, improving convenience, portability, and decreasing radiation exposure to both operator and patient [3].

On March 22, 2020, the British Orthopaedic Association (BOA) produced guidelines to help surgeons manage urgent orthopaedic conditions and trauma during the coronavirus pandemic, updating them on April 20, 2020 [4]. It was advised that patient contact be limited in order to reduce disease transmission and that staffing levels be decreased due to isolation, infection, and redeployment [5]. Both proved to be important steps to undertake for the protection of patients and staff as the pandemic evolved over the last two years. It did, however, require a rapid paradigm shift in organisation and senior decision-making processes within outpatient fracture management to ‘Get It Right First Time’ ultimately reducing hospital attendances and the risk of catching COVID [6,7].

Re-deployed referral patterns changed during COVID because patients with certain hand injuries increased as more patients fell while cycling or were injured in DIY-related accidents [6,8]. Practices evolved to adapt to non-operative management pathways by using Fluoroscan (Hologic, Inc., Marlborough, MA) in the hand clinic without compromising the standard of care by manipulating more fractures in outpatients rather than main theatres or day-case facilities. The cases were streamlined by using fluoroscopy in the clinic, which is required for formal imaging within the orthopaedic department [9].

This study aims to evaluate the effectiveness of Fluoroscan-assisted fracture management in orthopaedic hand clinics and whether this helps to avoid more complex treatments and enhances patient safety.

## Materials and methods

Regulations require employers in the United Kingdom (UK), including hospital trusts, to have formal written rules and protocols outlining the safe use of medical radiation. Breaches of the regulations are a criminal offence under the Health and Safety at Work Act of 1974.

There is a need to have well-trained personnel with appropriate equipment and systems in place to perform an optimised and safe medical procedure that involves patient and staff radiological exposure. These regulations are mentioned in the Ionising Radiations Regulations (1999), which relates to occupational exposures and medical equipment. All medical radiation exposures in the UK are governed by the Ionising Radiation (Medical Exposure) Regulations (2000) [IR(ME) Regulations 2000]. IR(ME) regulations apply to patients exposed to any medical radiation for diagnostic, therapeutic, medicolegal, research, health surveillance or screening purposes. It is required that medical exposures are justiﬁed in providing a net beneﬁt to the patient.

**Figure 1:**
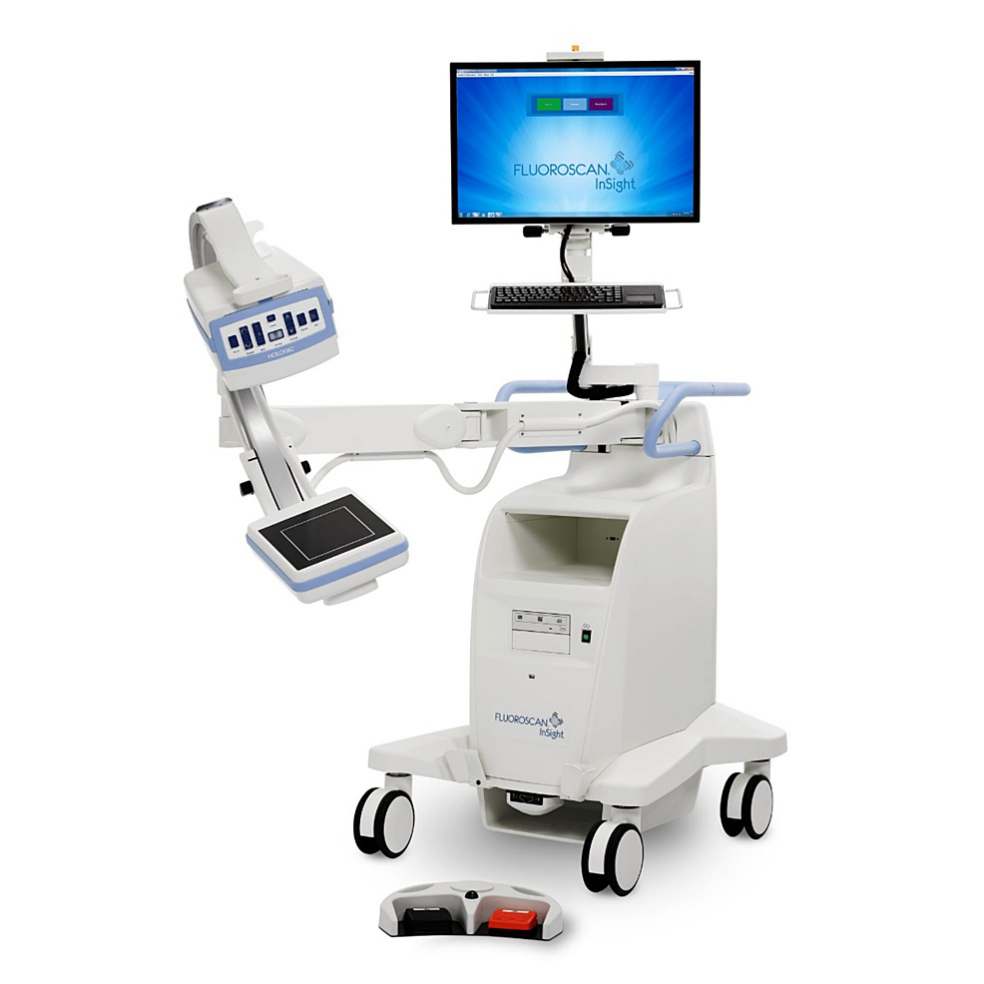
The Hologic Fluoroscan InSight used in our outpatient department.

Hologic Fluoroscan InSight has been used in this study. The Fluoroscan InSight consists of a microfocus X-ray tube and generator mounted at one end of a C-arm and a compact image intensiﬁer and TV camera mounted at the other (Figure 1). These are at a ﬁxed distance apart. To ensure that the X-ray source-to-skin distance is at a maximum, reducing the entrance surface dose to the patient, the intensiﬁer should be placed close to the patient. This also generates images of good quality, which are displayed on twin TV monitors. Using a smaller ﬁeld reduces the radiation dose to the patient, reduces the scattered dose to the operator and gives superior spatial resolution to the image.

The Fluoroscan component of the image intensiﬁer is free to move in all planes, the Fluoroscan image intensiﬁer also allows the operator to change the view angle by a few degrees and may reveal penetrations of joints by ﬁxations that are not visualised in the standard radiographic views. The images can be printed, or stored, on hard discs in a PC or picture archiving and communication system (PACS)-compatible format.

Although the Fluoroscan intensiﬁer is safe, IR Regulations (1999) govern the safety of employees and members of the public. It should be used in a controlled area in the clinic with obvious signs. Both the patient and the surgeon (operator) wear lead rubber aprons of 0.25 mm lead equivalence with hands kept out of the field.

The minimum X-ray source-to-skin distance is 30 cm, so, the intensiﬁer should be kept as close as possible to the target area. This decreases the entrance surface dose to the patient and improves image quality.

This study was conducted prospectively at Queens Burton Hospital for one year starting from April 2020, after the BOA guideline was introduced, until March 2021. During the COVID pandemic, Fluoroscan was moved to an appropriate room in the hand clinic identified by the Radiation Protection Officer (RPO) and the images produced were scanned into the notes as a record of the intervention. Appropriate training and safety checks were undertaken by the clinic staff under the supervision of the RPO, including audits of radiation usage. One surgeon, along with the plaster technician, performed the manipulation whilst a separate surgeon or trained nurse monitored the patient as per the Royal College of Surgeons standards for safe manipulations [10].

**Figure 2:**
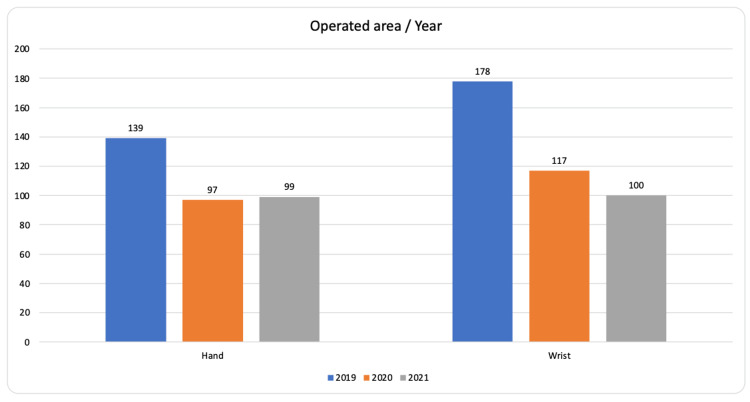
Chart compared the number of operated cases at Queens Burton Hospital in three sequential years (before, during and after COVID pandemic peak) – Hand and wrist cases were included.

The demographic data of the patients were collected for cases where the Fluoroscan had been used for screening or manipulations, and the mechanism of injury was noted, which included information about the bone fractured, type of intervention and required analgesia. The need for admission and further treatment was recorded and final outcomes were derived using established literature norms for radiographic and functional outcome assessment using an appropriate functional assessment tool, the QuickDASH scoring system. The outcomes of normal pre-COVID-19 practice, which have been recorded on an access database prospectively to keep track of when and where injuries were operated on within the hospital, were compared with the outcomes of this study (Figure 2). Data capture and analysis was facilitated using a Microsoft Excel (Microsoft® Corp., Redmond, WA) spreadsheet for analysis. Thirteen patients were excluded as the data was incomplete.

## Results

The study included 83 patients who attended the hand clinic during the study period and required a manipulation. Females represented 2/3 of the patients with 1/3 being the male gender. In 82% of cases, the fracture involved the dominant hand, the average age of all patients was 53.16 years, and most of them (68 of 83) were injured in their dominant arm (Table 1).

**Table 1:** Distribution of the demographics data and the fracture manipulation details

Demographic data		n (%)	
Sex			
	Male	27 (33%)	
	Female	56 (67%)	
Side			
	Right	68 (82%)	
	Left	15 (18%)	
Fracture manipulation details		n (%)	
Time interval between fracture and manipulation			
	Less than 24 h	30 (36%)	
	1-7 days	38 (46%)	
	8-14 days	11 (13%)	
	More than 14 days	4 (5%)	
Type of anaesthesia used			
	Haematoma block	16 (19%)	
	Penthrox	22 (26%)	
	Entonox	34 (42%)	
	Combined Block + Penthrox	10 (12%)	
	None	1 (1%)	
Operating person grade			
	Consultant	57 (69%)	
	Speciality doctor	26 (31)	
Radiology exposure (DAP) / Gy cm^2^			
	Less than 1	62 (74%)	
	From 1 to 2	17 (21%)	
	From 2 to 3	4 (5%)	
	More than 3	0 (0%)	
Reduction			
	Satisfactory	59 (71%)	
	Unsatisfactory and required surgery	19 (23%)	
	Accepted/Not fit for surgery	3 (4%)	
	Accepted/Patient refused surgery	2 (2%)	
Physiotherapy outcome for non-operated cases (64 cases) (QuickDash score)			
	Satisfactory (no residual disability)	60	
	Unsatisfactory (residual disability)	4	

Fifty-three cases were distal radial fractures following a simple fall (Table 2). Thirty patients had manipulations from ED minors and were fast-tracked into the fracture clinic, further reducing the need for reattendance. Thirty-eight cases were manipulated within seven days of injury, and the highest interval between injury and manipulation was 17 days. Three main methods of analgesia were utilised to facilitate the manipulation, including hematoma block, Penthrox, Entonox or a combination. Two-thirds of the manipulations were performed by upper limb consultants and the rest were done by senior upper limb trainees. The Dose Area Product (DAP) averaged 1.3 Gy cm^2^ with all cases being below the safe lower limit (less than 3) according to the radiation protection protocol implemented within the hospital (Table 1).

**Table 2:** Reduction outcome regarding each type of fracture *Unsatisfactory reduction not operated due to either not fit for surgery or patient refused surgery. Another trial of manipulation with Fluoroscan has been done for better alignment.

Fracture type		Total	Satisfactory	Unsatisfactory	Accepted*
	Distal radius	53	39	11	3
	Carpal/CMC	3	0	3	0
	Metacarpal	19	14	3	2
	Phalangeal fingers	8	6	2	0

A satisfactory fracture reduction was maintained by using the Fluoroscan in 59 cases over an average of six weeks. Reduction was not maintained in 24 with five being re-manipulated, three because the patients were not fit and two due to patient preference (Table 1).

The availability of theatres and case numbers reduced during the COVID-19 pandemic. It was no longer possible to staff regular day case lists for instance. The shortfall in theatre availability was met in part by being able to manipulate the patients initially in clinic to a better position which was maintained in 64 cases. Surgery was undertaken in 19 of cases (12 as day cases and seven at the main site) where the reduction could not be maintained at an average of 12 days post manipulation and 16 post injury.

There was a significant reduction in the demands on theatre by adopting this practice as shown in Figure 1, by almost one-third of the total number of operated cases before the COVID pandemic. Nearly all these cases eventually achieved a good to excellent score on QuickDASH after a physiotherapy follow-up, irrespective of the type of intervention. Table 2 shows the outcome in each fracture type.

## Discussion

During the COVID-19 pandemic, locating the Fluoroscan in the hand clinic helped reduce patient’s exposure to risk by decreasing footfall within the orthopaedic department, because they no longer had to track through the departments cyclically after having plaster change and then X-ray in the radiology department. Reducing the number of patients needing to go to the X-ray department was important during the pandemic as this is also where COVID patients were having chest imaging. Over a third of the cases were fast-tracked from the Emergency Department minors which were located within the Orthopaedic Department further reducing attendances and footfall.

Fluoroscan provides an effective way of reducing fractures through manipulation, with 77% of cases having achieved satisfactory results without the need for hospital admission and surgery. In 2007, a hand clinic in Derby observed 100 patients including fracture manipulations, joint screening, and guided injections. This was seen to facilitate diagnosis and effectively manage patients in the outpatient department, achieving a quicker and earlier outcome, sometimes in a single patient episode, providing immediate beneﬁts to all [11]. It does not seem to have been adopted as a service improvement nationally in part due to the cost of Fluoroscan machines but also the lack of a suitable room to house the machines. The lack of theatre staff made these machines available for relocation during COVID as they were no longer being used in theatres.

The use of Fluoroscan in the outpatient clinic reduced the need for formal X-rays by over 90%. In addition, the Fluoroscan provided improved imaging for the surgeon who could get more views with less radiation exposure to guide treatment and enable screening in comparison to the standard 2 views offered by conventional X-rays. In certain situations, such as during the evaluation of fracture-dislocations of the base of the little finger metacarpal (eight cases in our series) for adequate reduction, this even diminishes the need for a computed tomography scan which may equate to over 3000 seconds of screening [11].

Swindells et al. used the mini-C-arm in outpatient clinics and concluded that it has improved the patient pathway, was easy to use and produced high-quality images that can be stored digitally. Furthermore, it provided dynamic images and direction for interventions such as injections [9].

On the other hand, a study of 60 distal radius fractures was obtained to compare the quality of the fluoroscopic images obtained from a mini-C-arm to those from formal radiographs. Six orthopaedic surgeons and one radiologist reviewed the images. The study showed that the likelihood that further imaging is necessary to guide treatment decisions was higher by around twice for the mini-C-arm imaging group [12]. This was not the experience within this study where outcomes for injuries such as wrist fractures followed historical norms with 11 patients eventually having surgical intervention out of 53 (20%). Results in the literature show that for 100 fractures manipulated you can expect 10-20% to displace [13]. So, the quality of the manipulation achieved was likely to be comparable to those achieved in a theatre setting. However, the need for surgery depends on the configuration of the fractures with complex intraarticular and volar fractures being more likely to require surgical stabilisation [14].

## Conclusions

In conclusion, the COVID pandemic created an unprecedented situation that necessitated a change in practice due to a reduction in space, personal and theatre availability. Introducing Fluoroscan into the hand clinic enabled orthopaedic surgeons to treat more patients in a timely and safe fashion within the trust. This has become adopted as a permanent service improvement within the department, freeing up expensive theatre time to address the backlog in elective surgery.
